# On the Recent Advances in the Theory of Vision

**Published:** 1872-06

**Authors:** H. Helmholtz

**Affiliations:** The Royal Society of London


ART. II.—*On the recent Advances in the Theory of Vision. By* H. HELMHOLTZ, (*of the Royal Society of London.*)

Translated from *Les Annales d' Oculistique*, BY F. W. ABBOTT, M. D.

(*Continued.*)

When two circumstances are connected by an unvarying relation, one is the sign of the other. It appears to me that innumerable errors and many false theories depend upon this, that in the study of the perceptions, we have not up to this time distinguished with sufficient care between the notion of *sign* and that of *image.*

For it to be an *image* it is necessary that this representation should be of the same kind as the object represented; it is an image only upon this condition. A statue is the image of a man in so much as it imitates by its form, the form of the one whom it is intended to represent. Even when the question is concerning a statuette of reduced size, the dimensions of the original are not less always represented by certain dimensions.

A painting is the image of the original because it imitates, on the one hand, his colors by certain analagious colors, and on the other hand, because it imitates a part of his dimensions, those which it presents in perspective, by certain corresponding proportions.

The nervous irritations in our brain, and the representations in our consciousness may be images of what occurs in the external world, in so much as by their chronological order the representations imitate the chronological order of the facts, and in so much as they represent the relations of objects by the relations of the signs, so that the order of succession of the external objects is reproduced.

This relation between objects and the representations which we make of them, is evidently sufficient for our intellect for the task which it sets for itself, of seeking out the analogies which exist in the midst of the diversity which the external world presents, and re-uniting them to form from them ideas or laws. I will show in the third part of this exposition that this relation answers equally for all practical wants.

It is undoubtedly the case that uneducated persons, who are accustomed to repose implicit confidence in the testimony of their senses, are not the only ones who will scarcely be disposed to admit so great a want of accord between the qualities of the sensation and those of the object which occasions it; those who recognize the existance of illusions of the senses will not, perhaps, have less difficulty in declaring themselves convinced. Have not physicists hesitated a long time and made and exhausted all sorts of objections before admitting the identity between the rays of light and heat, whose essential difference appears to reside only in the nature of the sensation produced? If Goethe, as I have elsewhere stated, refused to accept Newton's theory of colors, did not this depend above all on the fact that he could not persuade himself that white, which produces the sensation of brightest light, could be composed of darker colors? This fact, discovered by Newton, was the germ of the new doctrine of the energies of the senses; his contemporary John Locke was likewise able to expound with perfect occuracy the principal propositions relating to the signification of the qualities perceptible by the senses. Here, manifestly is found the stone of stumbling for many persons, and for all that I nowhere encounter the contrary opinion formulated with sufficient precision for it to be possible to demonstrate its falsity. I think that this depends upon the fact that the causes of dissent rest upon reasons of a more fundamental order.

We remark, first of all, that it is necessary to be very careful not to confound to *seem* and to *appear*. The colors which bodies present appear in consequence of certain objective differences in the nature of the bodies; so, even from the scientific point of view they are not *vain appearances,* even though the manner in which they appear depends principally upon the nature of our nervous apparatus. It is only a *deceitful appearance* when we confound the normal appearance of one object with that of another. Now, this does not take place in the vision of colors: they have no manner of appearing which we may call normal, as it regards the manner in which they appear to the eye.

The principal difficulty here appears to me to lie in the notion of *property*. Everything becomes plain as soon as we are convinced that, in general, every property or quality of an object is, in reality, nothing else than the aptitude of this object to produce certain actions upon other objects. The action may be produced either between the analogous particles of the same body, whence result the differences of the state of aggregation, or, as chemical reactions, between the particles of two different bodies, or finally upon the organs of the senses, and it then manifests itself by sensations analogous to those which we are here considering. We give to such an action the name of *property*, when the reagent by which the effect is produced is implied without being expressed. When we speak of the solubility of a substance, we mean the way in which it acts in relation to water; when we speak of its weight, we understand the force which draws it to the earth; it is by the same right that we say that a substance is blue, because we imply, as useless to say, that we only have to do with the effect which it produces on a normal eye.

But since that which we call property is always a relation between two things, such an action evidently cannot depend solely upon the nature of the thing which acts; it necessarily depends also upon the nature of the thing upon which the effect is produced. Thus it would be nonsense to speak of the properties of light in itself, of those things which it might possess independently of every other object, and to attempt to rediscover these properties in the sensation of the eye. It is a logical contradiction to suppose such properties; nothing of the sort can exist; there can exist therefore no conformity between the sensation of colors and similar qualities inherent in light.

We think that these reflections have been imposed upon many profound thinkers for a long time; we find them clearly expressed by Locke and Herbart, and they are in accordance with the ideas of Kant. To make onesself master of this subject a remarkable faculty of abstraction would perhaps be necessary, while we have to do with notions whose evidence becomes, so to speak, palpable owing to the facts which we have exposed.

After this excursion into the domain of abstractions let us return to colors, and examine them in so far as they are the *sensual signs* of certain external properties pertaining either to light or to the bodies which reflect it. For a sign to be good, we demand first of all that it be constant, that is to say, that the same object should always accompany the same sign. Now we have already seen that even in this respect sensations of colors leave something to be desired. They are not entirely uniform in the retinal field; but the constant motion of the eyes shuns the rock here by the same process which has served to remedy inequalities in the sharpness of the retinal image. This way which we employ of looking at objects renders the defect less sensible.

In the next place we have seen that in consequence of fatigue, the intensity of the irritation may rapidly undergo considerable variations. Here also the constant motion of the eyes has generally the effect of spreading the fatigue equally over the whole retinal field; so that it rarely happens that well defined accidental images are formed, unless it is from very luminous objects, such as the sun or very brilliant flames.

Now, from a uniform fatigue of the whole retina the intensity of the colors which we look at varies almost the same for all, and fatigue produces simply the same effect as though the illumination decreased a little.

This leads us to study the variations which the visual images undergo, when the illumination of the objects varies.

We still encounter instructive facts here. We are able to see the objects of the external world under an illumination whose intensity varies in enormous proportions: the brightest solar light is 150,000 more intense than the light of the full moon. The color of the illumination may also sensibly change, whether we use the artificial illumination of flames, which always diffuse a more or less redish yellow light, or are in the greenish shadow of the foliage of trees, or in a room furnished with curtains and hangings of intense colors. The intensity and the color of light which illuminated bodies send to the eye varies evidently with the intensity and the color of the illumination. We know that the differences between the colors of objects proceed from the fact that different substances reflect and absort unequally the light of the different simple rays of the sun. Vermilion reflects, without sensibly enfeebling them, the rays whose wave-length is considerable, but it only reflects feebly all the other rays. It is for this reason that it appears to us of a red color, that of the rays which it reflects to the eye. If we illuminate it with a light which contains no red, it appears almost black.

As a result of this, the apparent color and intensity of illuminated objects ought to vary with the color and intensity of the illumination. Every day experience offers us a hundred examples of this fact, which furnishes to painters an important subject of study and upon which rest, many of their most brilliant effects.

But what interests us most in vision is to distinguish and to recognize the objects which surround us; especially if, from an esthetical or philosophical point of view, we sometimes turn our attention to the illumination. Now, the element which is constant in the color of an object is neither the intensity, nor the color of the light which it sends to the eye, but the relation between the intensities of the different colored elements of this light and the intensities of the corresponding elements in the illumination. This relation is nothing less than the expression of a constant property of the object.

In view of these theoretical considerations, we would think that it would be extremely difficult to appreciate the color of an object when the illumination varies. We see on the contrary, that in practice the appreciation of colors takes place with the greatest certainty, even without reflecting, and in the most varied conditions. Illuminated by the moon white paper is darker than black velvet in clear day light; nevertheless we never hesitate to recognize the paper as white and the velvet as black. Much more, if a gray object, illumined by the sun, reflects a light of the same color and perhaps also of the same intensity as a shaded white object, we have much more difficulty in recognizing this than the identity of the color of a white shaded paper and a white paper illumined by the sun. Gray seems to us specifically different from white; it is thus, when it constitutes the color of an object, because the surface of a body which reflects part of the light ought necessarily to be constituted otherwise than that of a body which reflects the whole of it. Nevertheless illumined gray may furnish the same impression to the retina as shaded white. Painters employ gray to represent white situated in the shade, and if they proceed correctly the object represented appears perfectly white. To persuade ones self experimentally of the identity of the color of gray and of white it is only necessary to strongly illumine, by means of a leas, a gray disc surrounded by a white back ground, so that the limits of the illumination correspond with those of the disc, which prevents the artificial character of the illumination from manifesting itself. Then the gray disc appears as white as the back ground.

We may admit certain, phenomena of contrast authorise it, that the illumination of the purest white furnishes us the standard for judging of the dark objects which surround it, because, in ordinary conditions, the intensity of all the colors deminishes uniformly when the illumination becomes more feeble, or when the fatigue of the retina increases.

For the extreme degrees of illumination, this uniformity of variation persists objectively, but does not re-appear in the sensations. When the illumination is blinding, the differences of intensity of the bright parts become scarcely perceptible to sensation: when the illumination is very feeble, it is the intensities of the darkest objects, on the contrary, which become imperceptible in their turn. Thus in the light of the sun, the colors of objects of mean intensity resemble more the colors of the brightest objects, while in moon light they approach those of the darker bodies. Painters profit by this difference to represent illumination by the sun and that by the moon. To express illumination by the sun, painters are obliged to brighten the tints of the objects of mean intensity, while to represent illumination, by the moon they darken the tint of these same objects. There is still another difference which rests upon sensation. When the intensity of the illumination increases, the impression of the red and of the yellow increases more rapidly than that of the blue. If we match, according to intensity, a blue paper and a red paper by day light, the equality of the intensity does not persist in different illuminations: in the sun the red seems more intense, while by the light of the moon or the stars the blue predominates. The same difference exists for the spectrum colors; painters also give a yellowish tint to their landscapes, illuminated by the sun, and a blueish tint to those which are designed to represent the effect of moon-light.

That which precedes shows to what a degree our appreciation of colors becomes independent of the absolute intensity of the illumination. So also, we are accustomed to abstract almost completely the influence exerted by the color of the illumination. We know that, to a certain degree, the light of candles is yellowish red in comparison with that of day; but to see how great the difference between these two light is, it is necessary to see them simultaneously, with the same intensity and one beside the other, as takes place in the experiment with colored shadows. Let us allow the light of a covered sky, that is to say the feeble light of day, or preferably the light of the moon to penetrate into a darkened room, and hold a white paper horizontally so that the light shall fall obliquely on the paper; we will let the light of a candle fall on the paper from another quarter, then a rod held perpendicularly to the paper gives two shadows; the one, where the light of day does not reach and which the candle alone illumines, is reddish yellow and seems such; the other, projected by the candle and where the light of day alone reaches, is white, but appears blue by contrast. The blue and the reddish yellow of the two shadows are the colors to which we give the name of white according as the dominant illumination is that of day or that of the candles. When these two colors are found side by side, they seem very different and even to some extent saturated. Nevertheless we never hesitate to recognize the white paper as such by candle light and to distinguish it from an orange colored paper.

What is more remarkable in this series of phenomena is, that we do not confound the color of a colored transparent covering with that of the objects situated behind it, and from this springs a series of very interesting phenomena of contrast. Still further, when we look through a green vail it may happen that white objects, the light from which is mixed with green by its passage through the vail, appear reddish from the formation of the accidental color of green which is red. This experiment shows to what a degree we separate the color of the objects themselves, from that of a transparent medium through which we view them.*

Changes of color analogous to those noted in the two last experiments are called *phenomena of contrast.* For the most part these are illusions relating to the color of objects and attributable to illy defined accidental images. That which is produced when we look successivily at colored objects we call *successive contrast* But the phenomena of contrast depend also in part upon the fact

*See a series of experiments of this this kind in my Physiological Optics, pp. 398-400; 404-411. (Pages 523-525; 530-538 of the French translation.)

that the habit of judging of the colors of objects from the relations of intensity and of color of the different objects seen simultaneously may deceive us, when the relations differ from those to which we are accustomed. It is this which takes place, for example, when we are in the presence of two sources of illumination, or of colored transparencies, when we think that we are in the presence of homogeneous conditions. Then is produced *simultaneous contrast.* In the experiment of the colored shadows, for example, the doubly illuminated back ground, which is the brightest of surrounding objects, gives us a false standard for white: compared with this white, the true white of one of the shadows, which is less intense, appears blue to us. What increases still more the effect of these contrasts is, that differences of sensation seem to us greater when they are clearly perceptible than in the contrary case. Now the differences between the colors which we see simultaneously are clearly perceptible in comparison with those which we only compare from memory; the differences between neighboring objects in the visual field are clearly perceptible in comparison with those between the colors of more remote objects. All this is far from being without influence. A number of the most varied circumstances, whose study in detail throws light upon the grounds upon which we judge of the colors of objects, forms a chapter which we must content ourselves with simply noticing here. This subject is, moreover, just as interesting as it regards the theory of painting as for the physiologist, because painters very often purposely exaggerate the natural phenomena of contast to produce in the mind of the spectator an impression of differences of intensity and of saturation greater than those of which the colors of their palettes enable them to obtain a representation.

We have come to the end of the theory of sensations. It follows from our research that the qualities of the visual sensations are nothing else than signs relating to certain qualitative differences either of the light or of the illumined objects, but do not posses an objective signification exactly corresponding; we have even found that these signs are far from presenting, with perfect exactness, constancy, this essential quality of a system of signs: All that we have been able to say in their favor is only that, *under the same conditions,* the same qualitative sensations are developed by the same objects. But in spite of all we find, finally, that this system of signs suffices to enable us, constantly and everywhere to recognize colors with an accuracy sufficient and even astonishing, if we consider the difficulties of this important task. In the midst of a constantly varying system of intensities and of colors, varying with the illumination, with the fatigue of the organ, with the part of the retina affected at the moment, we are able to disengage this constant quantity, the color of the object, which depends upon the invariable nature of its surface, and this operation does not demand long reflection; it is instantaneous and its result has for us the nature of evidence.

The inaccuracies and imperfections which we have found in the optical apparatus and in the retinal image are nothing in comparison with the incongruities which we have encountered in the domain of the sensations. We might say that nature is delighted in accumulating contradictions to overthrow entirely the theory of a pre-existing harmony between the external and the internal world.

Have we reached the end of our study? We think not; the problem being complicated it would appear that we ought not to attempt to discover how vision takes place. Nevertheless let us not reproach science with depopulating by a sterile criticism the beautiful domain of our senses; neither let us plant ourselves upon a pretended common sense to accord a greater confidence to the testimony of our senses than to the results of physiology.

It remains for us to study the notions of space. Let us see whether in this last chapter science will not finish by justifying the confidence which we give to the testimony of the senses.

(To be continued.)

